# Energy-Filtered High-Resolution Electron Microscopy for Quantitative Solid State Structure Determination

**DOI:** 10.6028/jres.102.002

**Published:** 1997

**Authors:** Z. L. Wang, D. van Heerden, D. Josell, A. J. Shapiro

**Affiliations:** National Institute of Standards and Technology, Gaithersburg, MD 20899-0001

**Keywords:** Al/Ti, composition-sensitive imaging, electron energy-loss spectrosocpy, energy-filtering, high-resolution electron microscopy, Ni/Ti

## Abstract

Energy-filtered (or selected) electron imaging is one of the future directions of high-resolution electron microscopy (HREM). In this paper, the characteristics and applications of energy-selected electron imaging at high-resolution for structure determinations are illustrated. It is shown that image contrast can be dramatically improved with the use of an energy filter. High-resolution chemical-sensitive imaging using ionization-loss electrons is demonstrated in studies of Ni/Ti and Al/Ti multilayer thin films. It is also shown that the spatial resolution of energy-selected ionization edge electron images is dominated by the signal-to-noise ratio. Experimental parameters which may be selected to improve the signal-to-noise ratio are discussed.

## 1. Introduction

Quantitative structure determination at atomic resolution is one of the major research fields of high-resolution electron microscopy (HREM). Traditional methods rely upon qualitative comparisons between experimental micrographs and image simulations based upon structural models, with the acceptability of the particular model being considered as improved if an image “match” is obtained for several members of a through-focal series [1]. Iteration between calculations and experimental images based upon least-squares refinement methods can significantly improve the accuracy of the quantification. These HREM images, however, are usually recorded without the use of an energy filter, so that both the elastically and inelastically scattered electrons contribute to the image. This makes quantitative data analysis difficult because only the elastic scattering of electrons can be accurately simulated using existing dynamical theories. An energy filter can remove all of the inelastically scattered electrons except those scattered by phonons, which typically suffer an energy loss less than 0.1 eV, much less than either the resolution of the filter or the energy spread of the emission source.

The ultimate goal of energy filtering is to remove the inelastically scattered electrons in the recorded image/diffraction pattern so that quantitative structural and/or chemical information can be obtained. The interaction between an incident electron and the atoms in condensed matter results in several inelastic scattering processes [2]. Thermal diffuse scattering (or phonon scattering) is the result of scattering from atomic vibrations in crystals. This process does not introduce any significant energy loss. Valence-loss excitations (or plasmon excitation for metals and semiconductors) arise from the transitions of electrons from the valence band to the conduction band and involve an energy loss in the range of 1 eV to 50 eV. This process is the result of collective excitation of the solid and is closely related to the electronic structure of the material. Atomic inner-shell ionization is excited by the incident electron, resulting in an ejected electron from the inner-shell state. This ionization process is characteristic of the corresponding element, and it can thus be used to quantitatively determine the chemical composition of the specimen. Bremsstrahlung is the result of a continuous energy-loss process generated by an electron penetrating into the specimen and undergoing collisions with the atoms in it, resulting in electromagnetic radiation. Finally, electron Compton scattering is the collision of the incident electron with a specimen electron, resulting in a broad energy-loss peak from a few tens of eV to several hundred eV wide, with higher energy losses at larger scattering angles.

In this paper, we first introduce a technique for performing energy-filtered (or selected) HREM using signals generated from elastic and inelastic excitation processes. Several merits of the energy-filtered HREM are shown using the images recorded from MgO cubes and Al/Ti and Ni/Ti metallic multilayers. It is demonstrated that image contrast can be significantly improved with the use of an energy filter. Composition-sensitive images using electrons with energy losses characteristic of the ionization edges are illustrated. Finally, some practical limitations on the energy-filtered HREM are discussed.

## 2. Energy Filtering in TEM

There are two methods for performing energy filtering in transmission electron microscopy (TEM). Both the techniques rely on the physical mechanism of electron energy-loss spectroscopy. One method uses a Castaing-Henry filter, which consists of two 90° magnetic prisms and a retarding electric field [3, 4]. The filter is located between the objective lens and the intermediate lens. The electrons are sent to a 90° electromagnetic sector where the electrons having different energies are dispersed. They are then reflected by an electrostatic mirror, after which a second 90° prism deflects the electron back onto the optical axis. A slit is placed before the intermediate lens and selects the electrons with specific energy losses. The Castaing-Henry filter is unsuitable for primary beam voltages greater than approximately 100 kV. This energy filtering can only be performed on a specially built TEM. A detailed introduction of this energy filtering system and its applications has been given by Reimer et al. [5–7]. The detection limit of this technique for chemical imaging has recently been considered by Berger et al. [8].

**Fig. 1:**
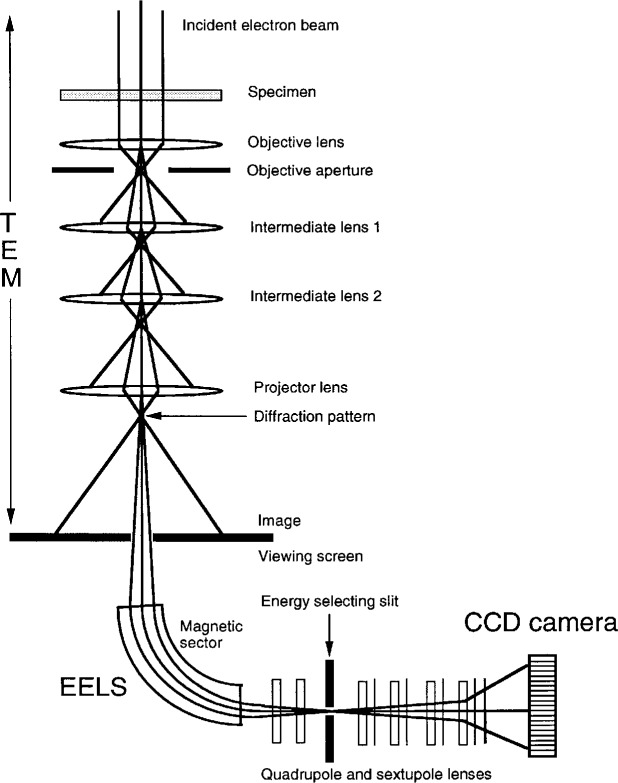
A schematic diagram showing the energy-filtering system attached to the NIST JEOL 3010 HREM.

The other energy-filtering method uses a parallel-detection electron energy-loss spectroscopy (PEELS) system attached to the bottom of a TEM [9, 10], schematically shown in Fig. 1. The system is composed of four components: TEM, electron energy-loss spectrometer (EELS), energy-filtering system and charge-coupled device (CCD) camera for digital data recording. The operation of the TEM is almost independent of the energy-filtering system, because the energy filtering occurs after the electrons have passed through all the lenses belonging to the TEM. The electrons are dispersed by magnetic sectors in the EELS, thus, electrons having different velocities (or energies) are focused on different positions in the plane of the energy-selecting slit. The energy-selecting slit selects electrons with a particular range of energy losses. A set of lenses then re-disperse the selected electrons to form the image (or diffraction pattern). The final image/diffraction pattern is recorded digitally using the CCD camera. This energy-selecting system can be fitted to any existing TEM without any modification to the electron optics. More importantly, energy filtering of high-resolution TEM images can be performed using this method. However, only a small portion of the transmitted electrons are allowed to go through the energy-filtering system because of the small size EELS entrance aperture needed to keep on-axis optical alignment. Thus, the signal-to-noise ratio is a limit of the technique.

**Fig. 2:**
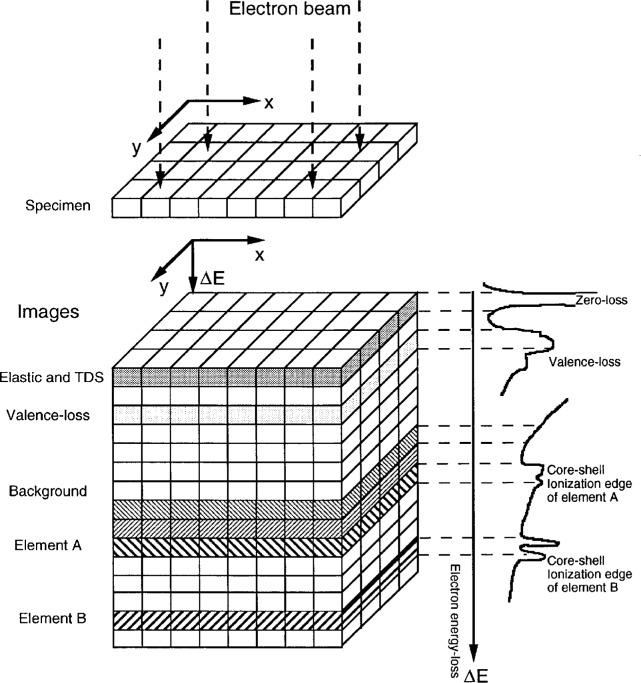
A schematic diagram showing energy-selected electron imaging in TEM. The energy-selected electron images corresponding to different characteristic energy loss features are shown. These can be used to extract useful structural and chemical information of the specimen.

With an energy filter, images (or diffraction patterns) formed by electrons with specific energy losses can be obtained (see Fig. 2). The energy-selected electron images can be simply illustrated using a three-dimensional data space, in which the *z*-axis is replaced by the energy loss of the electrons, and *x* and *y* are the real space coordinates of the two-dimensional image of the specimen. All of the inelastic scattering processes introduced above are observed in the EELS spectrum, as is shown schematically on the right-hand side of Fig. 2. The zero-loss (or 0-loss) peak is composed of elastically and thermal diffusely scattered electrons. The images/diffraction patterns produced by the elastically scattered and by thermal diffusely scattered electrons are incoherent. The low-loss region is dominated by valence excitations. However, as was discussed previously, the energy-selected image of valence-loss (or plasmon-loss for metals) electrons is not strongly composition sensitive because their scattering is closely related to the valence-band structure of the material. Atomic inner-shell ionization edges can be employed to form composition-sensitive images. The background observed in the EELS spectrum is produced by scattering processes of multiple valence losses, electromagnetic radiation and electron Compton scattering. In order to extract composition-sensitive images, the contribution made by these processes must be subtracted from the recorded data.

Only the inner-shell ionization signal directly reflects the concentration of the corresponding element in the specimen. To extract this information, two energy-selected images are recorded with the energy-selecting window placed at two pre-edge energy losses. Thus, the background image of typical shape *A*(*x*,*y*) exp[−*r*(*x*,*y*) Δ*E*] can be determined [9], where *A* and *r* are position-dependent constants to be determined by the local EELS Δ*E* spectrum, and is the electron energy loss. This background image will be subtracted from the image recorded when the energy-selecting window is positioned at the ionization edge. As the electron image reflects the projected structure of the specimen, the intensity variation in the energy-selected image, after the subtraction of the background, is proportional to the thickness-integrated concentration of the corresponding element. Therefore, the chemical-composition map is approximately proportional to the intensity ratio of background subtracted images recorded from two ionization edges. The reliability of this approximation will be discussed later.

The HREM experiments were performed using a newly installed JEOL 3010^1^ HREM (300 kV) with a point-to-point image resolution of 0.167 nm and ± 20° specimen tilt. The microscope is equipped with a windowless Ge energy dispersive x-ray spectrometer (EDS) which permits chemical analysis for both light and heavy elements from a region as small as a few nm, and a Gatan image filtering (GIF) system that allows both parallel-detection EELS and energy-selected imaging/diffraction. The microscope is also equipped with a scanning unit that permits backscattered, bright-field and dark-field STEM imaging, and x-ray chemical mapping. A resolution better than 2 nm can be achieved in the STEM mode using either bright-field or backscattering detectors. A CCD camera, placed at the end of the energy-filtering system, allows digital recording of electron images and diffraction patterns at 1024 pixels × 1024 pixels resolution. The recorded image can be processed using a variety of image processing software.

## 3. HREM of Elastically Scattered Electrons

**Fig. 3:**
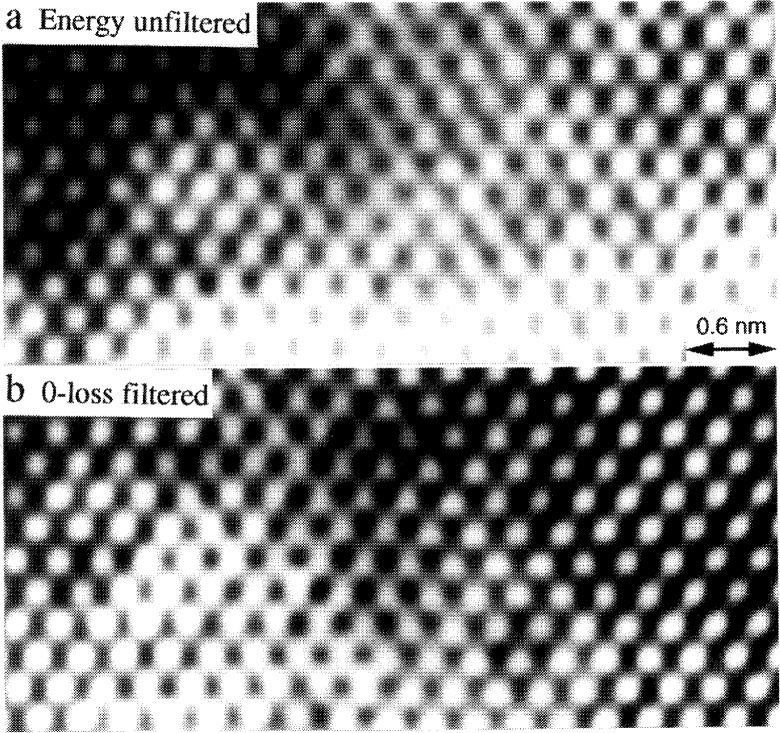
(a) Energy unfiltered and (b) zero-loss filtered [110] HREM images of a MgO cube, showing the increase of image contrast when the energy filter is applied. Energy window width was 5 eV, data acquisition time 2 s.

An important application of the energy filter is to form HREM images using those electrons which have not lost energy, i.e., purely elastically and thermal diffusely scattered electrons. Figure 3 shows a pair of [110] lattice images of a MgO cube recorded without (Fig. 3a) and with (Fig. 3b) energy filtering. The MgO cube is oriented along [110], so that the contrast variation across the image in the horizontal direction is due to the thickness variation of the MgO cube. It is apparent that the image contrast is dramatically improved in the 0-loss energy-filtered image. The merit of energy filtering is particularly pronounced when the specimen thickness is increased, since the probability of inelastic scattering is proportional to the specimen thickness.

**Fig. 4:**
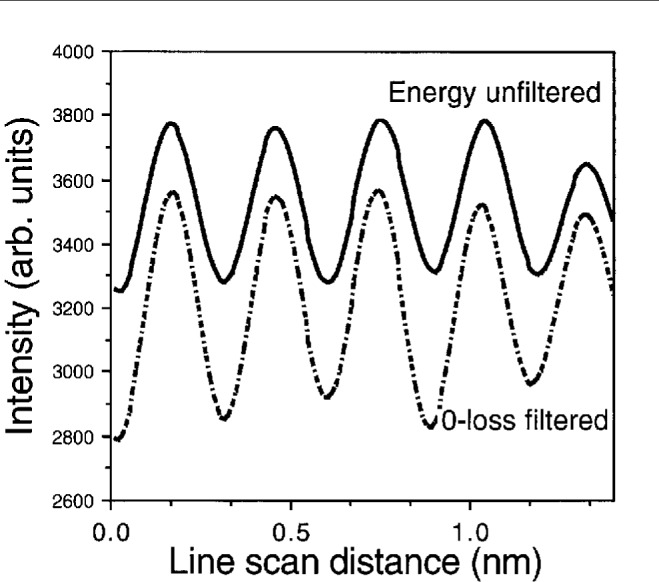
Intensity line scans across the images shown in Fig. 3.

To quantitatively evaluate the influence of inelastic scattering on the image contrast, Fig. 4 shows a comparison of intensity line scans across the images recorded with and without applying an energy filter. It has been found that the image contrast is improved by 50 % after applying the energy filter. This is a substantial improvement which benefits quantitative structure determination. Figure 4 demonstrates that the inelastically scattered electrons smear out image contrast because of chromatic aberration and inelastic angular distribution.

**Fig. 5:**
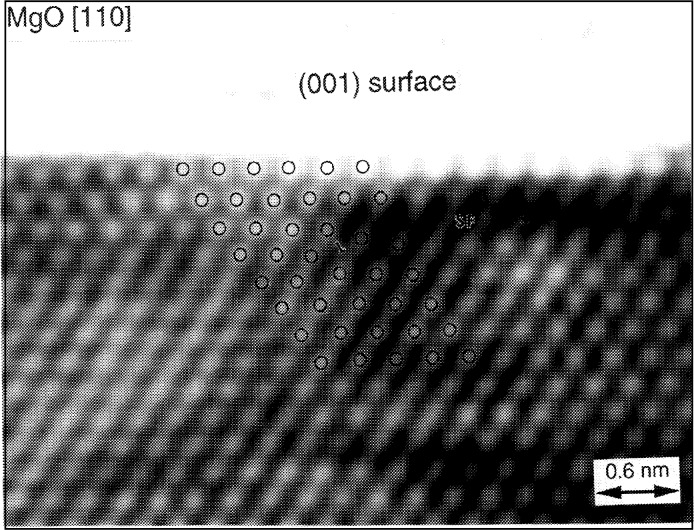
Zero-loss energy-filtered [110] HREM profile image of a MgO cube, showing the edge-on (001) surface and a stacking fault as well as a dislocation beneath the surface layers.

In HREM, atomic structures of crystal surfaces can be imaged if the surface is parallel to the incident beam, so that the arrangement of the projected atom rows can be directly imaged at the optimum resolution power provided by the microscope (0.167 nm for the microscope used here). This technique is known as surface profile imaging [1]. Figure 5 shows a zero-loss energy-filtered profile image of MgO(001) surface when the crystal is oriented along [110]. The observed intensity variation at the top surface layer results from Fresnel fringes [11]. The Mg atomic columns show bright contrast, and are indicated by circles in the figure. A stacking fault (SF) and its associated partial dislocation are seen at the third Mg layer from the surface. This structure is expected to be unstable for ionic crystals unless there are surface adsorbates, presumably O, O-H and C. Unfortunately, these adsorbates cannot be resolved in the HREM image because of their small scattering power. However, these adsorbates are probably present on the surface, since the specimen was prepared in air.

## 4. Composition-Sensitive Imaging of Energy-Selected Electrons

Determining lattice structure and atom types filling the lattices are two critical steps in quantitative structure determination. The crystal lattices can be determined reasonably well using HREM and various diffraction techniques. However, the determination of local chemistry at high spatial resolution is a more challenging task because HREM is not sensitive to the presence of light elements due to their weak scattering. Difficulties are also encountered when the difference between atomic numbers is small. Therefore, compositional imaging at high spatial resolution is vital for solving problems in materials science.

There are four basic methods for obtaining composition-sensitive images. In scanning transmission electron microscopy (STEM), inelastically excited signals, such as x rays and Auger electron emissions, are acquired sequentially as a function of electron scanning position [12, 13]. A two-dimensional display of the acquired signal intensity gives a distribution map of the corresponding element. Backscattered electrons in STEM (or scanning electron microscopy) give a composition-sensitive image because the atomic backscattering factor is proportional to the average atomic number *Z*. High-angle annular dark-field imaging (HAADF or “*Z*-contrast”) in STEM and high-angle hollow-cone dark-field TEM (HADF-TEM) provide chemical information about a specimen [14, 15] as the intensity of high-angle phonon-scattered electrons is also related to atomic number *Z*. Energy-selected electron images corresponding to atomic inner-shell ionization edges also allow chemical imaging in STEM [16, 17] and TEM [9, 18]. In this section, energy-selected electron images formed in TEM using different inelastic scattering signals are shown.

### 4.1 Valence-Loss Electrons

**Fig. 6:**
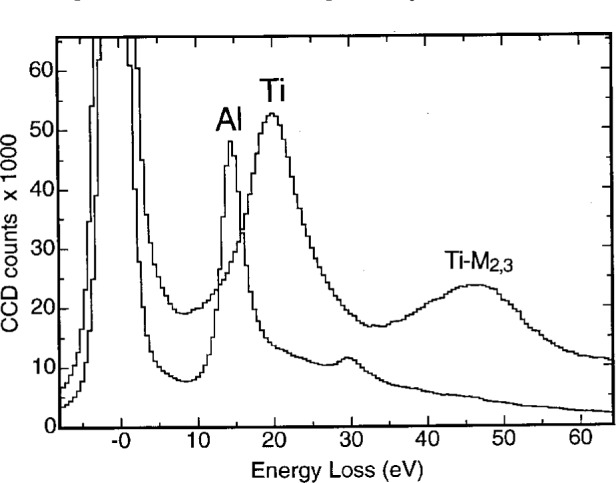
EELS spectra acquired from a Al/Ti multilayer specimen when the electron beam is positioned at Al and Ti layers, respectively. The shift of the plasmon peak results from the increase of free-electron density in Ti than in Al.

When an electron passes through a thin metal foil, the most noticeable energy loss is due to plasmon oscillations. For an ideal case in which the electrons can move “freely” in the electron sea, the system can be treated as an electron gas. This case is best represented by Al. The valence electrons can be considered as free electrons. Those negatively charged particles are mixed together with nuclei of positive charges, forming a solid state plasmon “gas.” The resonance frequency of this plasmon is directly related to the density of electrons in the solid. This simple plasmon model may also be adapted to describe the valence excitation of semiconductor materials, such as Si. For nonconducting materials, this plasmon model does not hold and the excitation is referred as valence band excitation. The plasmon oscillation frequency *ω*_p_, under the free electron approximation, is given by [19]:

$$\omega_{P}=e\sqrt{n_{e}/(m_{0}\varepsilon \varepsilon_{0})},$$

where *e* is the elementary charge, *m*_0_ is the electron rest mass, *n*_e_ is the density of free electrons, and *ε* is the dielectric constant of the material. Therefore, for two metals having different densities of free electrons, the plasmon peaks are expected to appear in different positions. Figure 6 shows a comparison of the plasmon-loss EELS spectra obtained from the Al and Ti layers of a metallic Al/Ti multilayer film. The Al plasmon peak is located at 15 eV and the Ti plasmon peak at 21 eV. If an energy-selecting window is set at 15 eV and then at 21 eV, the images formed will display the regions that correspond to Al and Ti, respectively.

**Fig. 7:**
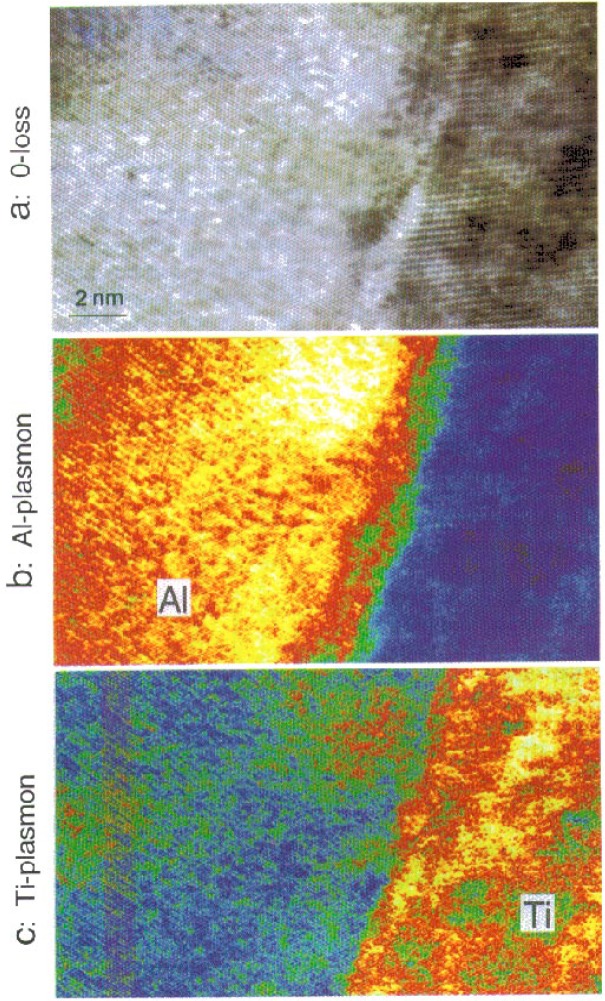
(a) Zero-loss, (b) Al-plasmon (Δ*E* = 15 eV) and (c) Ti-plasmon (Δ*E* = 21 eV) energy-selected HREM images of an Al/Ti (111) interface, showing the composition sensitivity. The lattice image can be formed by plasmon-loss electrons; the image intensity is influenced by phase and diffraction contrast.

Figure 7 shows energy-selected images of the 0-loss (Fig. 7a), Al-plasmon (Fig. 7b) and Ti-plasmon (Fig. 7c) electrons. It is apparent that the Al layer and Ti layers are shown in Figs. 7b and 7c, respectively. Notice that phase and diffraction contrast are apparent in the plasmon-loss energy-selected images because the small energy loss of one to two tens of eV does not affect the dynamical scattering behavior of the electron in the solid. This example clearly shows that plasmon-loss can, in some cases, be applied to get some useful local chemical information. It must be pointed out, however, that the energy-selected plasmon-loss image may not be sensitive to the local chemistry if there are strong overlaps in the plasmon-loss spectra. This is the case for most nonmetallic materials.

### 4.2 Inner-Shell Ionization Edge Electrons

**Fig. 8:**
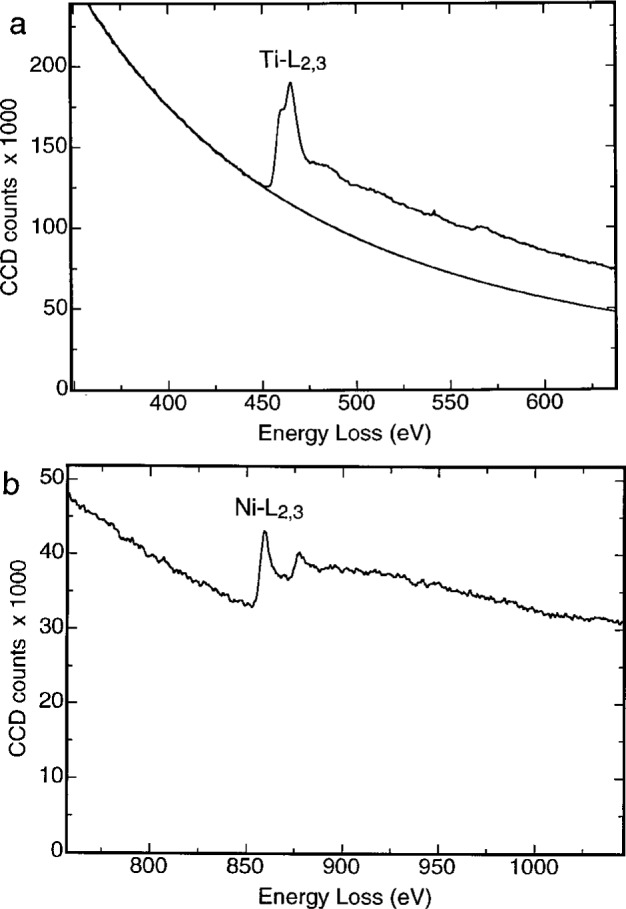
EELS spectra acquired from a Ni/Ti multilayer specimen when the elctron beam is positioned at (a) Ti and (b) Ni layers. The Ti-L_2,3_ and Ni-L_2,3_ edges are the fingerprints of Ti and Ni elements and can thus be used to form composition sensitive images.

Using the GIF system it is feasible to obtain chemical-sensitive images of a specimen at high spatial resolution, provided proper data processing is applied. Figure 8 shows EELS spectra acquired from the Ni and Ti layers of a metallic Ni/Ti multilayer specimen, exhibiting the Ti-L_2,3_ and Ni-L_2,3_ edges, respectively. These ionization edges are produced by the transitions of the inner-shell electrons to the free electron state. The transition is excited due to the energy and momentum transfers of the incident electron. The threshold of the edge is the binding energy of the electron. Thus, each edge is the fingerprint of the corresponding element, and it can be used to form a chemical map.

**Fig. 9:**
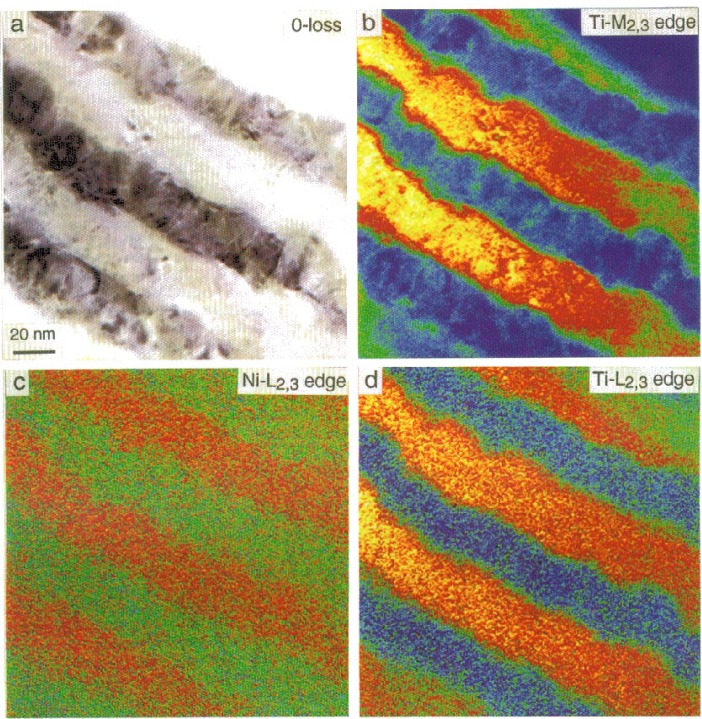
(a) Bright-field, (b) Ti-M_2,3_ edge (Δ*E* = 40 eV), (c) background subtracted Ni-L_2,3_ edge (Δ*E* = 854 eV) and (d) background subtracted Ti-L_2,3_ edge (Δ*E* = 455 eV) energy-selected HREM images of an Al/Ti (111) interface. Energy-window width 10 eV, data acquisition time 4 s for (b) and 15 s for (c) and (d).

Figure 9 shows a group of energy-selected cross-section images of a Ni/Ti multilayer specimen. The Ni and Ti layers are polycrystalline. The interface sharpness is not apparent from the bright-field image (Fig. 9a). The energy-selected image using the Ti-M_2,3_ edge (Figure 9b) clearly shows the distribution of the thickness integrated Ti concentration, and the Ti/Ni interface is reasonably sharp. The energy-selected image using the Ti-L_2,3_ edge (Fig. 9d) gives a similar result, although the signal-to-noise ratio is poorer due to lower signal intensity at the higher energy loss region. The energy-selected Ni-L_2,3_ edge image (Figure 9c), which shows the distribution of the Ni in the layer, is complimentary to the image recorded using the Ti-L_2,3_ edge.

**Fig. 10:**
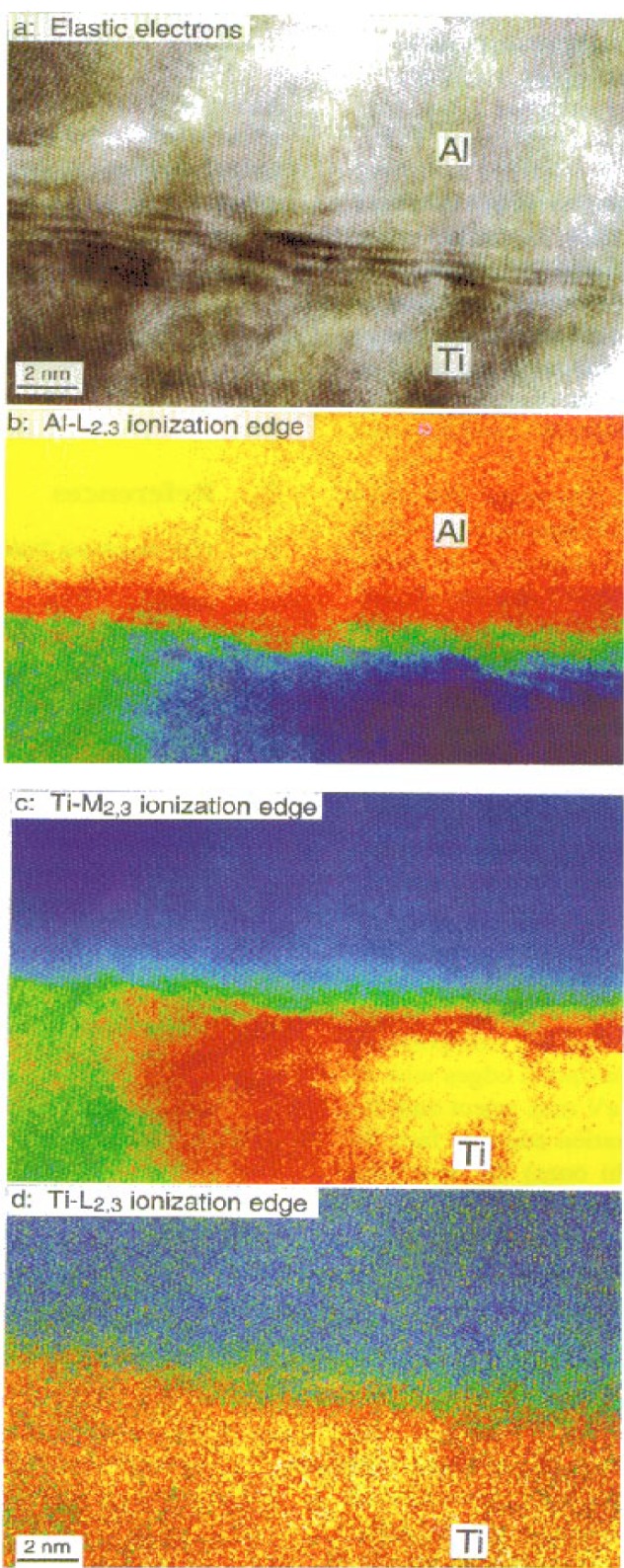
(a) Zero-loss, (b) background subtracted Al-L_2,3_ edge (Δ*E* = 73 eV), (c) Ti-M_2,3_ edge (Δ*E* = 40 eV) and (d) background subtracted Ti-L_2,3_ edge (Δ*E* = 455 eV) energy-selected HREM images of an Al/Ti (111) interface. Beam azimuth [10], energy-window width 10 eV, data acquisition time 5 s for (b) and (c) and 10 s for (d).

Figure 10a is a 0-loss HREM image of an Al/Ti interface, and Figs. 10b–d are the corresponding chemical images obtained using the electrons corresponding to different ionization edges of Al and Ti, respectively. The Al and Ti layers have a twin relationship, and the interface as seen in the 0-loss image (Fig. 10a) is not sharp. Diffraction contrast due to interface mismatch and stress makes the interface appear broad. Figure 10b is an energy-selected image of the interface using the Al-L_2,3_ edge electrons with energy losses from 73 eV to 83 eV, where the Al layer is clearly resolved. The change of contrast in the image reflects the variation of the projected (or thickness integrated) local density of Al. The image (Fig. 10c), recorded using the Ti-M_2,3_ edge located at an energy loss of 40 eV, shows the distribution of Ti. The image formed by the Ti-L_2,3_ edge, located at an energy loss of about 455 eV, also clearly reveals the location of the Ti layer (Fig. 10d). These images show that the Al/Ti interface is chemically sharp, and that the interdiffusion, if it exists, is limited to one to two atomic layers.

In contrast to the experimental results shown in Figs. 10c and 10d, theoretically the Ti-L edge is considered to be a more localized scattering than the Ti-M edge, thus a better spatial resolution is expected to be given by the image formed by the Ti-L edge. This discrepancy indicates that, as long as the selected electrons come from the inner-shell ionization with either lower or higher energy losses, the spatial resolution is mainly affected by the signal-to-noise ratio. Using ionization edge electrons it has been found that the best achievable spatial resolution is 0.4 nm [18].

## 5. Discussion

There are several possible ways of improving the signal-to-noise ratio in energy-selected compositional imaging. First, the use of a high-brightness field emission gun may improve the local current density. The GIF system allows only a 3 mm diameter area image, regardless of the microscope magnification, to pass through the spectroscopy and energy filtering systems. An FEG can enhance the total current passing into the energy-filtering system at higher image magnifications. Second, increasing the beam convergence may improve the local current density, but the spatial resolution may be reduced due to the non-parallel incident beam. Third, increasing data acquisition time is feasible for collecting lower resolution image, but the specimen drift becomes critical at high magnification. Fourth, ionization edges with lower energy losses are recommended, but caution must be exercised in the subtraction of the background image because of the irregular background shape due to multiple plasmon peaks. Finally, increasing the width of the energy-filter window can increase the signal intensity, but chromatic aberration limits the image spatial resolution, as illustrated below.

When forming images using electrons that have suffered an energy-loss Δ*E*,an additional focus shift of Δ*f*_c_ = *c*_c_ Δ*E*/*E*_0_ is introduced with respect to the 0-loss energy-filtered image due to chromatic aberration; here *c*_c_ is the chromatic aberration coefficient of the objective lens and *E*_0_ is the primary beam energy. Thus, the total defocus value is Δ*f* = Δ*f*_obj_ + Δ*f*_c_, where Δ*f*_obj_ is the defocus of the objective lens. Since *c*_c_ is typically about 2 mm, Δ*f*_c_ = 100 nm for an energy-loss window of Δ = Δ*e* = 15 eV. Therefore, the recorded image is the result of the superposed images of electrons with a defocus spread of 100 nm, reducing the spatial resolution. For general purpose applications, the width of the energy-selecting window is 10 eV to 20 eV.

It would appear that the optimum spatial resolution is obtained with the use of a small width energy window, provided the signal-to-noise ratio is large enough. Finally, it must be pointed out that the composition-sensitive image formed by the ionization edges with an energy window smaller than 20 eV may select only the near-edge structures of the ionization edge. In fact, for some elements (particularly light ones) the near edge structure is strongly influenced by the solid state effects [20]. Thus, the energy-selected ionization edge image, in general, should be referred to as a composition-sensitive image rather than a compositional image.

## 6. Conclusion

Energy-selected electron images can provide both structural and chemical information at high-resolution. Zero-loss energy selected HREM images exhibit significantly increased image contrast in comparison to the unfiltered images. High-resolution chemical imaging using inner-shell loss electrons in HREM has been demonstrated on Ni/Ti and Al/Ti multilayer thin films. Specimen chemical information can be provided by the Gatan image filtering system with an acquisition time shorter than 20 s, which is much shorter than the acquisition time using energy dispersive x-ray spectroscopy. It has been shown that the spatial resolution of the energy-selected ionization edge electron image is dominated by the signal-to-noise ratio; the signal localization, as long as it comes from inner-shell excitation, has little effect. Thus, an inner-shell ionization edge located in the lower energy-loss region, which has strong intensity, is recommended for chemical imaging.

## Footnotes

1 Certain commercial equipment, instruments, or materials are identified in this paper to foster understanding. Such identification does not imply recommendation or endorsement by the National Institute of Standards and Technology, nor does it imply that the materials or equipment identified are necessarily the best available for the purpose.
